# Assessing the Discriminatory Ability of Hemoglobin Concentration to Predict Iron Stores in Cambodian Females: A Systematic Review and Individual Participant Data Meta-Analysis

**DOI:** 10.1016/j.advnut.2026.100681

**Published:** 2026-06-15

**Authors:** Lulu X Pei, Colleen C Farrell, Stella C Mlewa, Cassandra Sauer, Kristija Sejane, Nathalie V Kirby, James McKendry, Christopher Charles, Jordie AJ Fischer, Tim J Green, Hou Kroeun, Jan Makurat, Aviva I Rappaport, Kyly C Whitfield, Frank T Wieringa, Allison I Daniel, Crystal D Karakochuk

**Affiliations:** 1 Human Nutrition, Faculty of Land and Food Systems, The University of British Columbia, Vancouver, BC, Canada; 2 BC Children’s Hospital and Women’s Health Research Institutes, Vancouver, BC, Canada; 3 Department of Anesthesiology and Pain Medicine, University of Toronto, Toronto, ON, Canada; 4 College of Nursing and Health Sciences, Flinders University, Adelaide, Australia; 5 Caring Futures Institute, Adelaide, Australia; 6 Helen Keller International, Phnom Penh, Cambodia; 7 Centre of Competence for Humanitarian Relief, Münster University of Applied Sciences, Münster, Germany; 8 Institute of Sustainable Nutrition, Münster University of Applied Sciences, Münster, Germany; 9 Department of Applied Human Nutrition, Mount Saint Vincent University, Halifax, NS, Canada; 10 Qualisud, University of Montpellier, CIRAD, Institut Agro, IRD, Université d'Avignon, Université de la Réunion, 34394 Montpellier, France; 11 Independent Consultant, Toronto, ON, Canada; 12 Department of Health Sciences, Faculty of Science, Amsterdam Public Health Vrije Universiteit Amsterdam, 1081 HV Amsterdam, the Netherlands

**Keywords:** anemia, Cambodia, discriminatory, ferritin, hemoglobin, iron, predict, receiver operating characteristic curve, females

## Abstract

Hemoglobin concentration is commonly used as a screening tool to guide iron interventions because it can be measured more quickly and easily using point-of-care devices than other, more direct measures of iron status (e.g., ferritin). However, anemia is not always caused by iron deficiency. Therefore, we aimed to assess the discriminatory ability of hemoglobin concentration to predict iron stores in Cambodian females of reproductive age, a population where iron deficiency prevalence is low and genetic hemoglobinopathies are common. We conducted a systematic review and individual participant data (IPD) meta-analysis that included 26 studies reporting on 11 databases (including *n* = 3497 females with available and eligible IPD that included hemoglobin, ferritin, and ≥1 inflammation biomarker measured in the same individual at the same time point). Receiver operating characteristic curve analyses were conducted to measure the area under the curve (AUC) and the 95% confidence interval (CI), representing the discriminatory ability of hemoglobin concentration to distinguish between individuals with or without iron deficiency (inflammation-adjusted ferritin <15 μg/L for nonpregnant females; unadjusted ferritin <30 μg/L for pregnant females). Random-effects models were used to estimate pooled effect sizes and 95% CIs. The pooled AUC value (95% CI) for nonpregnant females (9 studies; *n* = 3377) displayed “fair” discriminatory ability [0.77 (0.74, 0.79)] with high certainty evidence, and for pregnant females (2 studies; *n* = 120) displayed “poor” discriminatory ability [0.57 (0.47, 0.68)] with low certainty evidence. We conclude that hemoglobin is a “fair” predictor of iron status in nonpregnant Cambodian females; thus, it should be used with caution in guiding interventions such as blanket iron supplementation programs. More research is needed to understand if hemoglobin is an acceptable screening biomarker to guide interventions or justify higher doses of supplemental iron during pregnancy. The protocol was registered in PROSPERO (CRD42024578023).

**Statement of significance**

Global guidelines currently propose to use hemoglobin screening to guide iron interventions, even though the etiology of anemia is multicausal, with iron deficiency being only one, albeit important, factor. In this individual participant data meta-analysis of 26 studies (reflecting 11 datasets with *n* = 3497 females), we found hemoglobin to be only a “fair” predictor of iron status in nonpregnant females of reproductive age in Cambodia, and thus it should be used with caution in guiding iron interventions, such as blanket iron supplementation programs. More research is needed to understand if hemoglobin is an acceptable screening biomarker in pregnancy to guide interventions or justify the need for additional supplemental iron during pregnancy.

## Introduction

Anemia is a global health problem affecting almost one-quarter of the world’s population [1]. In 2011, it was estimated that ∼500 million nonpregnant females of reproductive age had anemia [2]. In nonpregnant females, anemia is defined as a hemoglobin concentration <120 g/L and is associated with an increased risk of fatigue, as well as reduced work capacity and productivity [3], which consequently hinders a country’s economic and social development [4]. Furthermore, if females have anemia during pregnancy, they are at a greater risk of adverse perinatal outcomes, such as postpartum hemorrhage, low birth weight, and small-for-gestational-age infants [5]. There is a long-standing assumption that ∼50% of anemia is due to iron deficiency in low-resource settings, which has been the impetus for global recommendations for blanket (untargeted) iron supplementation in many different settings and populations [[6], [7], [8]]. However, it is essential to consider that anemia can result from multiple underlying causes (e.g., infection, genetic hemoglobinopathies), indicating that a more nuanced approach may be necessary. For example, in a study including the analysis of anemia data from 23 countries, Petry et al. [9] found that the importance of iron deficiency in the etiology of anemia decreased with increasing prevalence of inflammation, with iron deficiency being associated with anemia in <25% of cases in populations with a high prevalence of inflammation.

In response to the significant health risks associated with anemia, especially during pregnancy, the WHO recommends that pregnant females consume daily iron and folic acid (IFA) supplements to reduce risk of maternal anemia, neural tube defects, puerperal sepsis, low birth weight, and preterm birth [6]. In line with this guidance, the Cambodian Ministry of Health has adopted its national nutrition policy recommending a daily IFA supplement (60 mg elemental iron and 400 g folic acid), and screening for anemia at the first antenatal care visit (ANC1; ∼8–12 wk of gestational age) by assessing hemoglobin concentration in a capillary blood specimen with use of a portable hemoglobinometer [10]. If diagnosed with anemia at ANC1, the guideline recommends that the daily dose of IFA be doubled (120 mg elemental iron and 800 g folic acid daily) [10]. Ideally, females would be screened for iron deficiency rather than anemia, but, unfortunately, there are no point-of-care devices currently available for ferritin assessment [11], and most health centers in Cambodia do not have the instruments required for the more complicated ferritin assay.

The primary aim of this study is to evaluate the current practice of using hemoglobin concentration as a screening tool to guide iron supplementation recommendations in a population where iron deficiency prevalence is low, and anemia and genetic hemoglobinopathies are common, such as in Cambodia [12]. We hypothesized that hemoglobin concentration would have poor discriminatory ability to predict iron stores in Cambodian females. We conducted a systematic review and individual participant data (IPD) meta-analysis to: *1*) assess the discriminatory ability of hemoglobin concentration to predict iron stores in Cambodian females of reproductive age; and *2*) determine whether the discriminatory ability of hemoglobin concentration differs by blood source (venous compared with capillary), analytical device (hemoglobinometer compared with hematology analyzer), or life stage (pregnant compared with nonpregnant females).

Ultimately, this work aims to generate new evidence to inform the discriminatory ability of hemoglobin to predict iron status in Cambodian females and discern whether hemoglobin should be used as a screening tool to guide iron supplementation practices in this population.

## Methods

This systematic review is reported in accordance with the PRISMA-IPD reporting guideline. The protocol was registered in PROSPERO (CRD42024578023) on 21 August, 2024. One amendment was submitted (28 October, 2025) to the protocol after the review commenced: the age range of eligibility was expanded from 18–45 to 15–49 y to align with the WHO definition of females of reproductive age [13].

### Electronic database searches

The search was conducted in Ovid Medline, Ovid Embase, and Scopus databases from their inception to 9 June, 2025. Searches included a combination of subject headings (e.g., medical subject headings in MEDLINE) and free-text keywords (.mp). The search included 3 broad themes: *1*) Cambodia, *2*) hemoglobin or anemia status, and *3*) ferritin or iron status. As the goal of the search was to identify records for an IPD meta-analysis, the search themes were broadly focused on the study population (Cambodian individuals) as well as the 2 main biomarkers (hemoglobin and ferritin) required. The detailed search strategy from Ovid Medline is provided in Supplemental Table 1. Randomized controlled trials, nonrandomized studies, cross-sectional studies, observational cohorts, case-control studies, or survey study designs were included, with no restriction on publication date. Unpublished studies, as well as those not reported in full, were also eligible.

### Eligibility criteria

Studies were considered eligible for inclusion if the population was Cambodian females of reproductive age (15–49 y). There were no restrictions on life stage (nonpregnant, pregnant, or lactating). Eligible studies were required to report on original IPD that included hemoglobin concentration (g/L), ferritin concentration (μg/L), and ≥1 of the 2 inflammation biomarkers [α-1 acid glycoprotein: AGP (g/L) or C-reactive protein: CRP (mg/L)] required to calculate inflammation-adjusted ferritin, measured in the same individual at the same time point. Original IPD was obtained by contacting the authors of eligible studies. If available, information on blood source collected for hemoglobin assessment (capillary compared with venous), analytical method used for hemoglobin assessment, age, life stage (e.g., pregnant compared with nonpregnant), and gestational age (if pregnant females were included) was also obtained. The primary outcome was iron deficiency status, using standard cutoffs of ferritin concentration <15 and <30 μg/L [11,14].

### Study selection and data extraction

**FIGURE 1:**
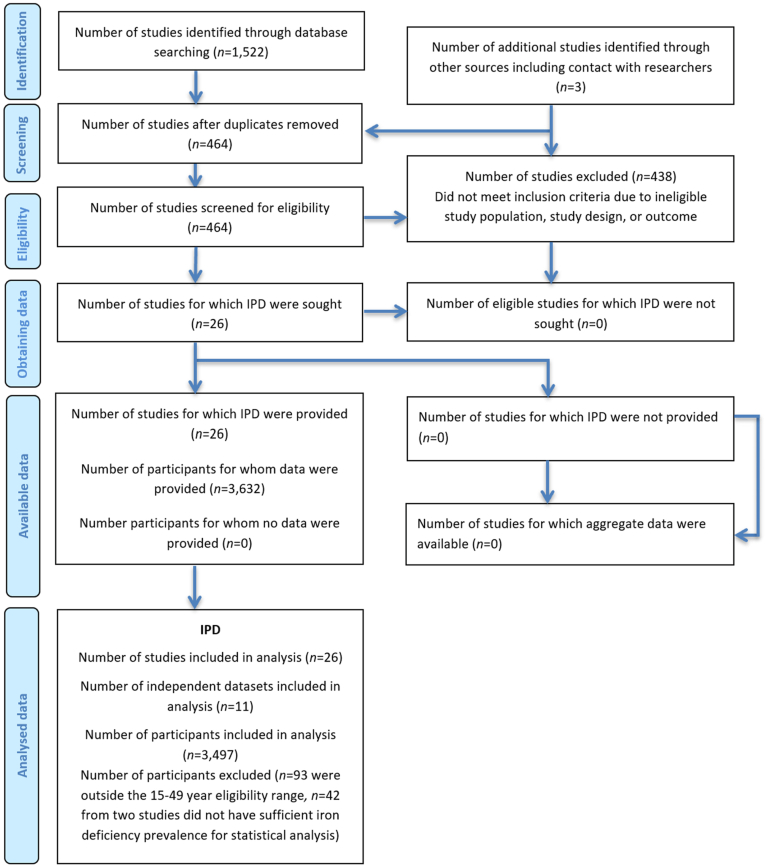
Flow diagram of identified and included studies. IPD, individual participant data.

All citations were uploaded to Covidence systematic review software (Veritas Health Innovation) to facilitate screening. Titles and abstracts were screened independently by 2 reviewers (CCF and SCM) against eligibility criteria. Full texts of all potentially eligible studies were screened independently by the same 2 reviewers (CCF and SCM). Studies that did not meet eligibility criteria were excluded, with reasons for exclusion recorded. During both title-abstract and full-text screening stages, any discrepancies were resolved by a 3rd reviewer (CS). The screening and selection processes are outlined in the PRISMA (IPD extension) flow diagram in Figure 1.

The authors of identified studies that reported on potentially eligible data were contacted by the principal investigator (PI) (CDK) to seek access to the original data. Ethical approval to share data was confirmed, and any required data-sharing agreements were secured.

### Assessment of data validity

All datasets were checked for completeness and accuracy by comparison with published data. For example, these checks included the comparison of study data published with study data received, such as sample size comparisons or iron deficiency prevalence rates, to verify the completeness of data received. Study authors were contacted for verification if discrepancies were identified.

### Data synthesis

Ferritin concentrations were adjusted for inflammation for nonpregnant individuals using CRP and AGP values following Biomarkers Reflecting Inflammation and Nutritional Determinants of Anemia (BRINDA) globally endorsed guidelines [15].

Receiver operating characteristic (ROC) curve analyses were conducted to measure the area under the curve (AUC)^ROC^ and the 95% confidence interval (CI), representing the discriminatory ability of hemoglobin concentration to distinguish between individuals with iron deficiency (inflammation-adjusted ferritin <15 μg/L for nonpregnant females [11] or unadjusted ferritin <15 μg/L and <30 μg/L for pregnant females [14]) and those without iron deficiency. The primary data synthesis approach was a 2-stage approach, where study-specific AUC (95% CI) values were determined first and then meta-analyzed to generate a pooled effect estimate. This approach was chosen a priori for the explicit preservation and visualization of between-study heterogeneity. However, given that some studies had no iron-deficient cases and therefore could not be included in the 2-stage analysis, sensitivity analyses using a one-stage approach, including all individuals, were also conducted. Random-effects models were used to estimate pooled effect sizes and 95% CIs. To account for the bounded nature of the AUC, analyses were conducted on the logit-transformed scale and then back-transformed to the original scale. The following thresholds were used to interpret AUC values: <0.7 was interpreted as having “poor”; 0.7 to <0.8 as “fair”; 0.8 to <0.9 as “good”; and ≥0.9 as “excellent” discriminatory ability [16]. The primary analysis included all eligible studies, separated by life stage (pregnant compared with nonpregnant). Further subgroup analyses by analytical device (hematology analyzer compared with hemoglobinometer) and by blood source (venous compared with capillary) were conducted. Forest plots were generated using R version 4.5.2 (R Foundation for Statistical Computing).

### Risk of bias assessment

Risk of bias (ROB) assessments were conducted using a modified version of the Quality Assessment of Diagnostic Accuracy Studies - Version 2 (QUADAS-2) tool with hemoglobin concentration compared against inflammation-adjusted ferritin as the current reference standard for assessment of iron status [17]. This modified QUADAS-2 tool is included in Supplemental Table 2, with the following categories: participant selection, hemoglobin measurement, ferritin as the reference standard, and flow and timing. When sufficient data were not available, we liaised with trial investigators as needed to obtain missing information. We also planned to assess availability bias, if IPD were obtained only from a subset of studies from which data were requested. Because some of the study team members were authors or coauthors on identified eligible studies in this IPD, to avoid real or perceived conflict of interest, we recruited additional study team members outside of the PI’s research team with expertise in the conduct of systematic reviews to assist with the ROB assessment on the included studies (NVK, AID).

### Certainty of evidence

As per the Grading of Recommendations Assessment, Development, and Evaluation (GRADE) Book for grading the certainty of evidence around the use of tests [18] experienced study member outside of the PI’s research team (AID) conducted GRADE assessments for each study group and subgroup in close consultation with the study team. The certainty of evidence started at high, and the evidence was evaluated based on 5 domains for rating down certainty (to moderate, low, or very low), including ROB, indirectness, inconsistency, imprecision, and publication bias.

## Results

**FIGURE 2:**
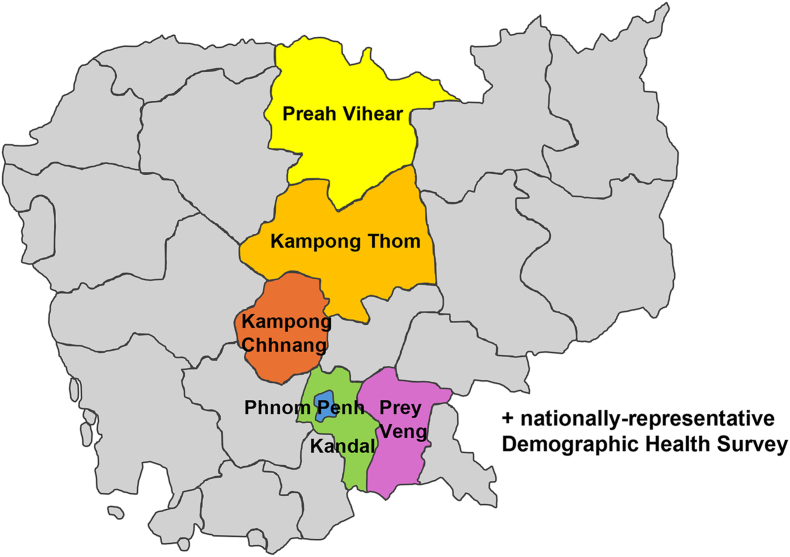
Map of Cambodian provinces and regions with included data.

We identified 461 unique studies through electronic searches and an additional 3 studies through other sources, including contact with researchers (total *n* = 464) (Figure 1). Of these, a total of 438 were excluded [*n* = 332 based on ineligibility via title and abstract screening; *n* = 101 because of the wrong outcomes (i.e., did not include measures of hemoglobin, ferritin, and ≥1 of the 2 inflammation biomarkers), *n* = 1 because of an incorrect study design, *n* = 3 because of the wrong patient population, and *n* = 1]. Twenty-six records met the inclusion criteria and were included in the analysis, which included publications (*n* = 21), conference abstracts (*n* = 2), and clinical trial registries (*n* = 3). Table 1 provides a summary of the 11 independent datasets from these 26 identified records (IPD for all identified studies were obtained), as well as the key publication of the dataset (year and 1st author) and other publications identified in the search [12,[19], [20], [21], [22],^23^,^24^,[25], [26], [27], [28]]. Geographically, the included IPD were representative of 6 provinces in Cambodia, with the addition of 1 nationally representative survey (Figure 2).

**TABLE 1:** Summary of characteristics of 11 included datasets (including a total of *n* = 3539 individuals; *n* = 3497 with eligible data for inclusion in IPD meta-analysis)^1^.

Author/y of key publication	No. of participants (*n*)	Region or province	Study design	Study population	Other publications from dataset^1^
Charles et al. 2011 [19] 2	35	Kandal	Longitudinal trial	Nonpregnant females >16 y	
Charles et al. 2015 [20] 3	173	Kandal	Cross-sectional	Nonpregnant females 16 y and older	
Fischer et al. 2023 [21]	480	Kampong Thom	Double-blind, 3-arm, placebo-controlled noninferiority trial	Nonpregnant females of reproductive age (18–45 y)	
Gallant et al. 2021 [22]	334	Kampong Thom	Double-blind, 4 parallel-arm randomized controlled dose-response trial	Mother (18–45 y) whose most recent pregnancy was normal, with a singleton infant born without complications	
Karakochuk et al. 2015 [12]	450	Prey Veng	Cross-sectional	Females of reproductive age (18–45 y, *n* = 420 nonpregnant, *n* = 30 pregnant)	Karakochuk et al. 2015 [29] Michaux et al. 2019 [30] Williams et al. 2020 [31]
Karakochuk et al. 2017 [23]	807	Kampong Chhnang	2 × 2 factorial, double-blind, placebo-controlled randomized trial	Anemic nonpregnant females of reproductive age (18–45 y with Hb concentration ≤117 g/L based on HemoCue finger-prick blood sample)	Cochrane et al. 2020 [32] Karakochuk et al. 2017 [33] Pei et al. 2021 [34] Pei et al. 2023 [35] Steele et al. 2019 [36] Williams et al. 2020 [31]
Makurat et al. 2016 [24]	217	Phnom Penh	Cross-sectional	Nonpregnant nulliparous females aged <31 y	Makurat et al. 2019 [37]
Mlewa et al. 2025 [25]	90	Prey Veng	Cross-sectional	3–8 mo pregnant with a singleton fetus	
Rappaport et al. 2017 [26]	326	Preah Vihear	Parallel, 3-arm, randomized controlled trial	Nonpregnant females of reproductive age (18–49 y) with mild-to-moderate anemia (Hb 80–119 g/L based on HemoCue capillary blood sample)	
Whitfield et al. 2017 [27]	175	Prey Veng	Double-blind, 3 parallel-arm randomized controlled efficacy trial	Nonpregnant female aged 18–45 y with ≥1 child aged 12–59 mo	
Wieringa et al. 2016 [28]	452	National	Nationwide, cross-sectional survey	Females 15–49 y (*n* = 445 nonpregnant, *n* = 7 pregnant)	

Abbreviations: Hb, hemoglobin; IPD, individual participant data.

1 References for conference abstracts (*n* = 2) and clinical trials (*n* = 3) registries are not included in the table. Williams et al. 2020 [31] is listed twice in the table, as it uses data from both Karakochuk et al. 2015 [12] and Karakochuk et al. 2017 [23].

2 Only a subset of participants from this dataset were included in our review (i.e., those in the control group with data available for ferritin and C-reactive protein) as the other participants did not meet our eligibility criteria for inclusion. In addition, those outside of our age eligibility window (15–49 y) were excluded.

3 Participants outside of our age eligibility window (15–49 y) were excluded.

### Anemia and iron deficiency prevalence

Ferritin and hemoglobin concentrations, as well as iron deficiency and anemia prevalence, are summarized for each dataset, including nonpregnant (Table 2) and pregnant individuals (Table 3).

**TABLE 2:** Ferritin and hemoglobin concentrations in IPD datasets including nonpregnant individuals^1^.

	Charles et al. 2011 [19] (*n* = 35)	Charles et al. 2015 [20] (*n* = 173)	Fischer et al. 2023 [21] (*n* = 480)	Gallant et al. 2021 [22] (*n* = 334)	Karakochuk et al. 2015 [12] (*n* = 420)	Karakochuk et al. 2017 [23] (*n* = 807)	Makurat et al. 2016 [24] (*n* = 217)	Rappaport et al. 2017 [26] (*n* = 326)	Whitfield et al. 2017 [27] (*n* = 175)	Wieringa et al. 2016 [28] (*n* = 445)
Unadjusted ferritin (μg/L)	84.0 [69.0, 102.0]	39.9 [25.5, 68.6]	80.7 [43.2, 116.8]	59.7 [36.0, 93.1]	90.8 [59.2, 142.3]	40.0 [17.8, 83.2]	34.5 [17.6, 62.1]	62.5 [36.7, 97.7]	73.4 [49.4, 107.4]	87.6 [56.6, 136.1]
ID, <15 μg/L	0 (0)	26 (15)	22 (5)	24 (7)	8 (2)	169 (21)	47 (22)	30 (9)	2 (1)	10 (2)
Inflammation-adjusted ferritin (μg/L)^2^	—3	—3	62.1 [35.0, 93.6]	46.9 [30.2, 73.4]	74.7 [45.7, 110.3]	30.8 [13.7, 57.6]	29.7 [16.3, 56.8]	59.6 [33.5, 87.0]	64.1 [43.8, 87.9]	55.5 [37.9, 75.9]
ID, <15 μg/L	—	—	33 (7)	32 (10)	8 (2)	221 (27)	52 (24)	34 (10)	2 (1)	21 (5)
Probable ID, <30 μg/L	—	—	95 (20)	82 (25)	39 (9)	396 (49)	109 (50)	68 (21)	22 (13)	69 (16)
Hemoglobin, measured with an automated analyzer (g/L)	128.7 ± 14.0^4^	—	129.0 ± 11.8^4^	—	124.5 ± 10.9^4^	115.9 ± 13.3^4^	—	117.7 ± 11.1^4^	124.3 ± 10.9^4^	—
Anemia, <120 g/L	8 (23)^4^	—	80 (17)^4^	—	124 (30)^4^	468 (58)^4^	—	164 (50)^4^	52 (30%)^4^	—
Hemoglobin, measured with a HemoCue (g/L)	—	121.3 ± 13.5^4^	—	118.0 ± 13.4^4^	122.0 ± 13.3^5^	107.2 ± 9.4^5^	124.6 ± 10.6^4^	109.1 ± 8.5^5^	—	120.5 ± 13.0^5^
Anemia, <120 g/L	—	76 (44)^4^	—	179 (54)^4^	172 (41)^5^	807 (100)^5^	59 (27)^4^	325 (100)^5^	—	187 (42)^5^

Abbreviations: ID, iron deficiency; IPD, individual participant data.

1 Total *n* = 3412 individuals from 10 datasets; *n* = 3377 with eligible data for inclusion in IPD meta-analysis. Values are median [IQR], mean ± SD, or *n* (%).

2 Ferritin concentrations were adjusted for inflammation based on α-1 acid glycoprotein and C-reactive protein values, as per Biomarkers Reflecting Inflammation and Nutritional Determinants of Anemia (BRINDA) guidelines [11].

3 Ferritin concentrations were not adjusted due to a lack of correlation between ferritin and C-reactive protein values, as per BRINDA guidelines [11].

4 Blood source was venous.

5 Blood source was capillary.

**TABLE 3:** Ferritin and hemoglobin concentrations in IPD datasets including pregnant individuals^1^.

	Karakochuk et al. 2015 [12] (*n* = 30)	Mlewa et al. 2025 [25] (*n* = 90)	Wieringa et al. 2016 [28] (*n* = 7)
Unadjusted ferritin (μg/L)	71.9 [25.7, 100.7]	35.5 [16.7, 65.9]	70.6 [41.6, 123.8]
ID, <15 μg/L	3 (10)	22 (24)	0 (0)
Probable ID, <30 μg/L	9 (30)	39 (43)	1 (14)
Hemoglobin, measured with an automated analyzer (g/L)	111.7 ± 10.2^3^	107.7 ± 10.2^3^	—
Anemia^2^	13 (43)^3^	44 (49)^3^	—
Hemoglobin, measured with a HemoCue (g/L)	107.9 ± 12.7^4^	—	111.1 ± 18.7^4^
Anemia^2^	17 (57)^4^	—	4 (57)^4^

Abbreviations: ID, iron deficiency; IPD, individual participant data.

1 Total *n* = 127 individuals from 3 datasets; *n* = 120 with eligible data for inclusion in IPD meta-analysis. Values are median [IQR], mean ± SD, or *n* (%).

2 Based on the WHO trimester-specific thresholds for hemoglobin [38]: <110 g/L for first and third trimesters; <105 g/L for the second trimester. Trimester data were not available for Karakochuk 2015 and Wieringa et al. 2016 [28] datasets; a hemoglobin concentration <110 g/L was used to define anemia in pregnancy in these 2 datasets.

3 Blood source was venous.

4 Blood source was capillary.

Among nonpregnant females (*n* = 3412), anemia prevalence within each included dataset (based on hemoglobin <120 g/L) ranged from 17% to 58%; iron deficiency prevalence (based on inflammation-adjusted ferritin <15 μg/L) ranged from 1% to 27%. Overall, among all nonpregnant females with anemia, only 24% (*n* = 329/1397) had iron deficiency anemia (hemoglobin <120 g/L and inflammation-adjusted ferritin <15 μg/L), and the remaining 76% of anemic nonpregnant females were iron-replete.

Among pregnant females (*n* = 127), 71% had available data on gestational age, and the mean ± SD gestational age was 24.4 ± 7.0 wk. Anemia prevalence within each included dataset (based on WHO trimester-specific hemoglobin thresholds [38], or <110 g/L if trimester data not available) ranged from 43% to 57%; iron deficiency prevalence (based on unadjusted ferritin <15 μg/L and <30 μg/L) ranged from 0% to 10% and 14% to 43%, respectively. Overall, among all pregnant females with anemia, 49% (*n* = 30/61) had iron deficiency anemia (hemoglobin <110 g/L and unadjusted ferritin <30 μg/L), and the remaining 51% of pregnant females with anemia were iron-replete.

### ROB assessment

We used a modified QUADAS-2 tool to assess the quality of the included studies (Supplemental Figure 1). Eight of the 11 (73%) included studies [12,[14], [15], [16], [17],^21^,^32^,^35^] had low ROB in all domains (i.e., participant selection, hemoglobin measurement, ferritin as the reference standard, and flow and timing). The ROB in the participant selection domain was deemed unclear for 1 study [39], because it could not be determined if a consecutive or random sample of participants was enrolled into the study. One study [19] was considered as having a high ROB for participant selection because participants were recruited ∼2 mo after completing a 1-mo course of iron supplements as part of a previous study. Furthermore, another study [28] was considered as having a high ROB for the flow and timing domain because correspondence with authors indicated that there was a notable delay between blood draws for hemoglobin and ferritin measurements (typically ≥1 wk). The certainty of evidence around the use of tests was assessed using GRADE for each study group and subgroup analysis (Table 4). Overall, the ROB across studies was low, and GRADE rankings varied from “very low” to “high” certainty in the 7 study groups or subgroups (explanations for downgrading are detailed in Table 4).

**TABLE 4:** GRADE assessment for each study group and subgroup.

N of studies (*n* of participants)	Factors that may decrease certainty of evidence					Certainty	Pooled AUC (95% CI)
	Risk of bias	Indirectness	Inconsistency	Imprecision	Publication bias		
Nonpregnant (inflammation-adjusted ferritin <15 μg/L)							
9 (3,377)	Not serious^1^	Not serious	Not serious^2^	Not serious	None	⊕⊕⊕⊕⊕ High^1^^,^^2^	0.77 (0.74, 0.79)
Pregnant (unadjusted ferritin <15 μg/L)							
2 (120)	Not serious^1^	Serious^3^	Not serious	Very serious^4^	None	⊕◯◯◯ Very low^1^^,^^3^^,^^4^	0.58 (0.45, 0.70)
Pregnant (unadjusted ferritin <30 μg/L)							
2 (120)	Not serious^1^	Serious^3^	Not serious	Serious^5^	None	⊕◯◯◯ Low^1^^,^^3^^,^^5^	0.57 (0.47, 0.68)
Subgroup: blood source = capillary blood specimen							
4 (1998)	Not serious^1^	Not serious	Serious^6^	Not serious	None	⊕⊕⊕◯ Moderate^1^^,^^6^	0.66 (0.63, 0.70)
Subgroup: blood source = venous blood specimen							
8 (2932)	Not serious^1^	Not serious	Not serious^2^	Serious^7^	None	⊕⊕⊕◯ Moderate^1^^,^^2^^,^^7^	0.77 (0.74, 0.80)
Subgroup: analytical method = hemoglobinometer							
7 (2722)	Not serious^1^	Not serious	Serious^6^	Serious^8^	None	⊕⊕◯◯ Low^1^^,^^6^^,^^8^	0.69 (0.66, 0.72)
Subgroup: analytical method = analyzer							
5 (2208)	Not serious^1^	Not serious	Serious^6^	Serious^7^	None	⊕⊕◯◯ Low^1^^,^^6^^,^^7^	0.77 (0.74, 0.80)

Abbreviations: AUC, area under the curve; CI, confidence interval; GRADE, Grading of Recommendations Assessment, Development, and Evaluation.

Explanations for each GRADE assessment are as follows:

1 Not serious: most information was from studies at low risk of bias for each of the 4 domains in the QUADAS-2 tool [17].

2 Not serious: there was inconsistency driven by 1 study (Whitfield et al. 2017 [26]), but sensitivity analysis with the removal of that study did not change the effect estimate and 95% CIs, and statistical heterogeneity (I^2^) in the sensitivity analysis was 0%.

3 Serious indirectness: there was a low prevalence of iron deficiency within and across the studies.

4 Very serious imprecision: the 95% CI crossed multiple thresholds of AUC, from no discrimination to “fair” as per study definitions [16].

5 Serious imprecision: the 95% CI crossed a threshold of AUC, from no discrimination to “poor” as per study definitions [16].

6 Serious inconsistency: point estimates and 95% CIs from individual studies differed in terms of their AUC values.

7 Serious imprecision: the 95% CI crossed a threshold of AUC, from “fair” to “good” as per study definitions [16].

8 Serious imprecision: the 95% CI crossed a threshold of AUC, from “poor” to “fair” as per study definitions [16].

### ROC curve analyses

**FIGURE 3:**
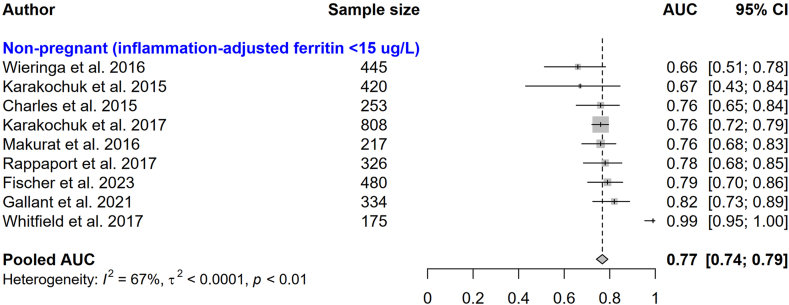
Forest plot displaying dataset-specific and pooled AUC values for the discriminatory ability of hemoglobin concentration in predicting iron status among nonpregnant Cambodian females. Data from Charles et al. [19] (*n* = 35) were not depicted because of a very low iron deficiency prevalence in the study population; therefore, it was not statistically possible to generate estimates for these data. Data from Charles et al. [20] include unadjusted ferritin concentrations because no association was observed between ferritin and C-reactive protein concentrations (thus, adjustment of ferritin is not recommended). AUC, area under the curve; CI, confidence interval.

A forest plot summarizing study-specific and pooled AUC values among the nonpregnant cohort is displayed in Figure 3. Nine datasets were included in the meta-analysis, as 1 dataset [19] had no iron deficiency cases; therefore, it was not statistically possible to generate estimates for these data. Consequently, in total, 9 datasets (including 3377 nonpregnant females) were included in the meta-analysis. For the 3 studies that reported hemoglobin measured in the same individual using both an automated hematology analyzer and a HemoCue hemoglobinometer [12,23,26], the former was selected and presented, as it is considered the reference-standard assessment method [38]. The pooled AUC value (95% CI) for nonpregnant females was 0.77 (0.74, 0.79) with high-certainty evidence (Table 4). There was considerable heterogeneity between studies (*I*^2^ = 67%, τ^2^ < 0.01, *P* < 0.01), with study-specific AUC values ranging from 0.66 to 0.99. Given the observed heterogeneity, an additional sensitivity analysis was conducted, removing the outlying result from the Whitfield 2017 database [27], which resulted in a comparable pooled AUC value (95% CI) of 0.76 (0.74, 0.79).

**FIGURE 4:**
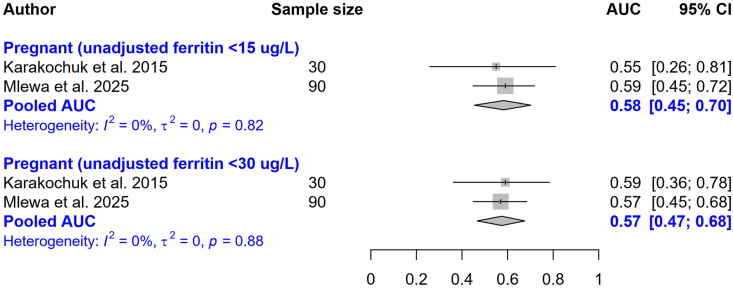
Forest plot displaying dataset-specific and pooled AUC values for the discriminatory ability of hemoglobin concentration in predicting iron status among pregnant Cambodian females. Data from Wieringa et al. [28] (*n* = 7) were not depicted because of a very low iron deficiency prevalence in the study population; therefore, it was not statistically possible to generate estimates for these data. AUC, area under the curve; CI, confidence interval.

Among the pregnant cohort, 2 of the 3 available datasets were included in the meta-analysis because, as noted above, in 1 dataset [28], there were no iron deficiency cases, and thus it was not statistically possible to generate estimates for these data. Therefore, in total, 2 datasets (including 120 nonpregnant females) were included in the meta-analysis. For the 1 study that reported hemoglobin measured in the same individuals using both an automated hematology analyzer and a HemoCue hemoglobinometer [12], the former was selected and presented, because it is considered the reference-standard assessment method [38]. The pooled AUC value (95% CI) for pregnant females using the ferritin cutoff of 15 μg/L was 0.58 (0.45, 0.70), but had very low certainty of evidence (Table 4), meaning that we have little confidence in these findings. The pooled AUC value (95% CI) for pregnant females using the ferritin cutoff of 30 μg/L was 0.57 (0.47, 0.68) and had low certainty of evidence (Figure 4; Table 4).

### Subgroup analyses

**FIGURE 5:**
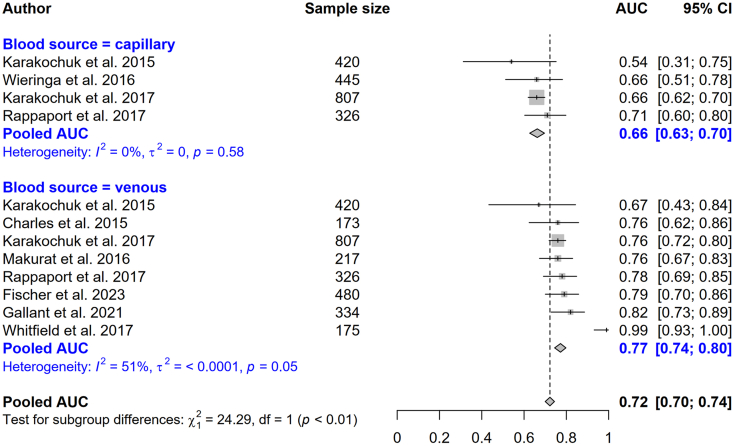
Subgroup analysis by blood source among nonpregnant Cambodian females. Forest plot displaying AUC values for the discriminatory ability of hemoglobin concentration in predicting iron status (deficiency defined as inflammation-adjusted ferritin <15 μg/L). AUC, area under the curve; CI, confidence interval.

**FIGURE 6:**
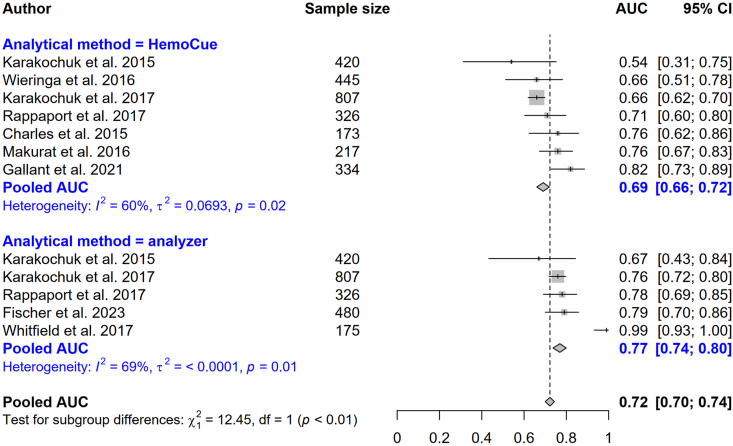
Subgroup analysis by analytical method among nonpregnant Cambodian females. Forest plot displaying AUC values for the discriminatory ability of hemoglobin concentration in predicting iron status (deficiency defined as inflammation-adjusted ferritin <15 μg/L). AUC, area under the curve; CI, confidence interval.

Among nonpregnant females, the pooled AUC value (95% CI) for hemoglobin concentration measured in capillary blood (from a finger-prick capillary blood specimen) was 0.66 (0.63, 0.70), compared with 0.77 (0.74, 0.80) for hemoglobin measured in venous blood (*P* < 0.01; Figure 5). Both estimates had moderate-certainty evidence (Table 4). When comparing analytical methods, the pooled AUC value (95% CI) for hemoglobin concentration measured via an automated hematology analyzer was 0.77 (0.74, 0.80), compared with 0.69 (0.66, 0.72) for hemoglobin measured via a point-of-care hemoglobinometer (*P* < 0.01; Figure 6); however, the certainty of evidence was low (Table 4). Subgroup analyses by measurement approach for the pregnant cohort were limited, given the small number of available studies (Supplemental Figure 2).

### Sensitivity analyses

A 1-stage meta-analysis approach including studies with no iron-deficient cases yielded a pooled AUC (95% CI) of 0.66 (0.64, 0.68) for the nonpregnant cohort, indicating “poor” discrimination ability. For the pregnant cohort, the pooled AUC value (95% CI) was 0.60 (0.48, 0.72) using a ferritin cutoff of <15 μg/L and 0.58 (0.48, 0.69) using a ferritin cutoff of <30 μg/L; both results indicate “poor” discrimination ability.

## Discussion

Our systematic search identified 11 independent datasets with available and eligible data for this IPD meta-analysis; we successfully obtained all 11 datasets from the authors, which provided data for an overall total of *n* = 3497 Cambodian females of reproductive age (15–49 y). The included IPD in this meta-analysis were geographically representative of 6 provinces in Cambodia and included data from 1 nationally representative survey.

In the nonpregnant cohort, we found high certainty of the evidence (9 studies; *n* = 3377) that hemoglobin concentration is a “fair” predictor (pooled AUC = 0.77) of iron status in Cambodian females of reproductive age. We speculate that heterogeneity between studies may be due to the inherent low prevalence of iron deficiency in this population [12,40], the differences in prevalence rates of anemia and iron deficiency across individuals in each of the study groups and subgroups, as well as the fact that anemia is often not caused by iron deficiency in this population [12]. It has been well-established that anemia in Cambodian females of reproductive age is more commonly caused by genetic hemoglobin disorders, which are autosomal recessive mutations that alter the structure and/or function of hemoglobin, and can cause anemia regardless of iron status [12,41]. It is important to appreciate that anemia is not a disease, but rather a condition that is reflective of its causes. Ultimately, these factors may have contributed to some of the observed imprecision in our pooled AUC estimates; however, we believe that it is unlikely that a larger or more generalizable sample of nonpregnant females in Cambodia would increase the certainty of these estimates, given these probable reasons for the imprecision.

In the pregnant cohort, we are less certain about the discriminatory ability of hemoglobin concentration to predict iron status in this population. We identified only 3 studies with available data, but only 2 had a sufficient number of iron deficiency cases to be included in the IPD meta-analysis. Therefore, we found very low certainty of evidence (2 studies; *n* = 120) that hemoglobin concentration is a “poor” predictor (pooled AUC = 0.57) of iron status (ferritin <30 μg/L) in pregnant Cambodian females. There was very serious imprecision noted among these 2 studies, where the 95% CI of the pooled AUC crossed multiple thresholds, from no discrimination to “fair” (0.47–0.68) [16], and serious indirectness (differing prevalence rates of iron deficiency across the 2 studies). In addition, gestational age for females included in 1 of the 2 included studies [12] was not known, which may explain the large variability in the 95% CI of the pooled AUC estimate. It is well-established that hemoglobin concentrations fluctuate across trimesters, as a result of the changing physiological processes that occur during pregnancy (e.g., second-trimester blood volume expansion [42]). This has justified the establishment of trimester-specific thresholds for hemoglobin during pregnancy [43]. There is a clear need for more studies with a larger or more generalizable sample of pregnant females, with known gestational age, to ascertain the utility of measuring first-trimester hemoglobin as a predictor of iron stores during pregnancy in Cambodia and beyond. However, the challenge also remains with the lack of established trimester-specific thresholds for ferritin (which have yet to be determined), and the uncertainty about whether ferritin should be adjusted for inflammation during pregnancy (as of current, the BRINDA adjustments are only recommended for nonpregnant females) [16,25].

We were also interested in subgrouping studies by the analytical device used for hemoglobin measurement (automated analyzers compared with point-of-care hemoglobinometers) and the blood source that was collected (venous compared with capillary specimens), because recent work has highlighted critical issues relating to inaccuracies in hemoglobin measurement using single-drop capillary blood specimens [[44], [45], [46]]. In our subgroup analysis based on 8 studies (including *n* = 2932 nonpregnant females), we found moderate-certainty evidence that hemoglobin, when measured in venous blood, was a “fair” predictor of iron status (pooled AUC = 0.77), and that it performed more optimally than hemoglobin when measured in single-drop capillary blood (pooled AUC = 0.66). This is likely due to the issues of measurement error in single-drop capillary blood, which is typically caused by operator issues (e.g., “milking the finger”) and variability in collection protocols and methods (e.g., timing between collection and testing, humidity) [47,48]. These findings align with current literature indicating that venous blood is the preferred blood source for accurate and reliable hemoglobin measurement. The latest WHO guidance now primarily recommends the collection of venous blood for measurement on automated hematology analyzers with high-quality control measures [1,46]; however, the potential for use of pooled capillary blood as an alternative to single-drop capillary blood remains an important research question to be answered [44].

From a policy and programming perspective, we were also interested in assessing the proportion of females in our IPD cohort who had anemia without iron deficiency, and consider what implications or consequences this might have in the Cambodian context. Our data suggest that iron supplementation programs that were based on a hemoglobin screen-and-treat approach would likely be providing unnecessary iron supplements to ∼76% of nonpregnant females with anemia in our study (as only 24% of nonpregnant females with anemia in our cohort had iron deficiency anemia). This has important implications because it results in an unnecessary allocation of valuable resources (e.g., cost of capsules and distribution), as well as potential adverse consequences of providing supplemental iron to those who do not need it (e.g., gastrointestinal side effects, oxidative stress, altered gut microbiome [49,50]). For pregnant individuals, our data suggest that a hemoglobin screen-and-treat approach would result in ∼50% of pregnant females with anemia in our study being advised to take additional iron supplements (according to the current supplementation policy in Cambodia, that would mean doubling the original dose of iron, from 60 to 120 mg elemental iron/d), when in fact, they are already iron-replete. This is even more worrisome for pregnant females with genetic hemoglobin disorders, because they are already at risk of excess iron accumulation (iron overload) as a result of altered iron homeostasis [51,52]. In pregnant females with hemoglobin disorders (and consequential iron overload), unnecessary and excess supplemental iron (120 mg elemental iron/d) may increase risk of impaired placental function and cause harm to other critical organs (e.g., liver, pancreas) [53].

It would be ideal to screen individuals for ferritin (a more direct measure of iron status) rather than hemoglobin to determine if any intervention (e.g., iron supplementation) is warranted. However, ferritin assessment is challenging in most rural health centers or settings, and no point-of-care devices yet exist. For most ferritin assays and analyzers, a plasma or serum specimen is required, which involves the collection of venous blood and more complex processing, transport, and storage requirements. Until research in this field advances further, we are left with the challenges and limitations of using hemoglobin concentration to guide iron interventions.

Advantages of this work include the inherent strengths of an IPD analysis, including enhanced quality and quantity of relevant data, more sophisticated and consistent statistical analysis across datasets, and more powerful aggregate-level meta-analysis of the selected outcomes. We successfully sourced all 11 datasets identified through our systematic literature search, representing diverse geographic and demographic contexts. Collaboration with the original dataset owners allowed us to verify data completeness and ensure more reliable, transparent results. We applied the BRINDA inflammation adjustments to ferritin concentrations across datasets for all nonpregnant individuals as per global consensus [15]. Lastly, our data segregation by life stage (pregnant compared with nonpregnant females) and subgroup analyses enhanced the accuracy of our study findings by comparing results across these heterogeneous groups (e.g., some of which had differing hemoglobin thresholds for anemia diagnosis).

We acknowledge some limitations of our work. First, the classification model for AUC determination is based on a ranking of positive and negative cases and may be overly optimistic and/or unreliable in the case of an imbalance in the dataset. We acknowledge that this may be applicable to our analyses, particularly for the study groups or subgroups with a low number of absolute cases of iron deficiency; therefore, we raise some caution in the interpretation of our pooled AUC estimates in these cases. Cambodia represents an interesting epidemiologic context in which iron deficiency prevalence is low despite a high burden of anemia, largely due to the substantial presence of genetic hemoglobinopathies and other non–iron-related causes of anemia. These features limit the generalizability of our findings to populations where iron deficiency is more common or where anemia is primarily driven by nutritional causes. However, similar contexts do exist outside Southeast Asia. For example, in Sierra Leone, other types of hemoglobinopathies are prevalent (e.g., sickle cell), and studies have also shown a similar low iron deficiency prevalence among females [54]. As such, our findings have relevance for comparable populations in other geographical settings [9]. Lastly, we also recognize our limited data available for pregnant individuals in Cambodia, which hindered our ability to assess the discriminatory ability of hemoglobin to predict iron stores in this study group.

In conclusion, it appears that hemoglobin is a “fair” predictor of iron status in nonpregnant females of reproductive age in Cambodia and should be used with caution in guiding interventions such as blanket iron supplementation. Global WHO guidelines have also confirmed that ferritin is a more accurate biomarker of iron stores than hemoglobin, and should be used to diagnose iron deficiency in apparently healthy individuals [43]. In settings where resources permit, clinicians and public health programs should prioritize measuring ferritin, alongside inflammation biomarkers such as AGP and CRP, to allow for appropriate adjustment when making decisions about iron supplementation [43]. We also reiterate that anemia is not a disease, but rather a condition that is reflective of its causes; therefore, the causes of anemia should always be first discerned before any interventions are considered. More research is needed to understand if hemoglobin is an acceptable screening biomarker to guide interventions, such as the need for additional iron supplements during pregnancy.

## Author contributions

The authors’ responsibilities were as follows – CDK: conceived the project, provided study oversight, conducted the systematic search strategy, and primarily responsible for the final content; LXP, CCF, SCM, CS, KS, CDK: developed the overall research plan; CS, CDK: conducted the systematic search strategy; CCF, SCM, CS: screened articles for eligibility; LXP: analyzed the data and performed the statistical analyses; CC, JAJF, TJG, HK, JM, AIR, KCW, FTW, CDK: contributed data; CCF, NVK, AID: performed risk of bias and GRADE assessments; LXP, CCF, SCM, CS, KS, AID, CDK: contributed to the first draft of the manuscript; and all authors: reviewed, contributed to, read, and approved the final manuscript.

## Data availability

Data and/or analytical code will be available upon reasonable request to the corresponding author (CDK) with approval by the study coauthors who have primary ownership of each dataset.

## Declaration of Generative AI and AI-assisted technologies in the writing process

The authors declare that no generative AI or AI-assisted technologies were used in the writing of this manuscript.

## Funding

LXP, CCF, and SCM are supported by a University of British Columbia Four Year Doctoral Fellowship. LXP and CCF are supported by a Canadian Institutes of Health Research (CIHR) Doctoral Award. CDK is a Michael Smith Foundation for Health Scholar and a CIHR Canada Research Chair Tier 2 in Micronutrients and Human Health. NVK is supported by the Muscle, Aging, (In)Activity, and Nutrition Laboratory. None of the sponsors played any role in the study’s conduct, nor had any restrictions regarding the submission of the report for publication.

## Conflict of interest

The authors report no conflicts of interest.
