# Supplementary material for: Assessing the Discriminatory Ability of Hemoglobin Concentration to Predict Iron Stores in Cambodian Females: A Systematic Review and Individual Participant Data Meta-Analysis
Source: Adv Nutr. 2026 Jun 15;17(7):100681. doi: 10.1016/j.advnut.2026.100681 (PMC13355708; doi:10.1016/j.advnut.2026.100681)

**Supplementary Online Content**

**Supplementary Table 1** Detailed search strategy and results

**Supplementary Table 2** Modified QUADAS-2 risk of bias tool

**Supplementary Figure 1** Subgroup analysis among pregnant Cambodian women (blood source and analytical method)

**Supplementary Figure 2** Risk of bias (ROB) assessment of included studies

**Supplementary Table 1** Detailed search strategy and results^1^

| **#** | **Search** | **Results from 10 June 2025** |
| --- | --- | --- |
| 1 | Cambodia/ | 4,079 |
| 2 | (Cambodia or kampuchea or khmer or Phnom Penh).tw,kf. | 5,678 |
| 3 | 1 or 2 | 6,558 |
| 4 | Hemoglobins/ | 75,806 |
| 5 | H?emoglobin*.mp. | 276,632 |
| 6 | Anemia, Iron-Deficiency/ | 12,616 |
| 7 | exp Anemia/ | 183,005 |
| 8 | Ferritins/ | 23,147 |
| 9 | Pregnancy Complications, Hematologic/ | 10,394 |
| 10 | (anemia or anemic or apoferritin* or ferritin* or isoferritin*).tw,kf. | 185,110 |
| 11 | iron status.tw,kf. | 6,610 |
| 12 | iron stores.tw,kf. | 4,298 |
| 13 | or/4-12 | 509,135 |
| 14 | 3 and 13 | 240 |

^1^ From Ovid MEDLINE (R) and Epub Ahead of Print, In-Process, In-Data-Review & Other Non-Indexed Citations, Daily and Versions <1946 to June 9, 2025.

**Supplementary Table 2** Modified QUADAS-2 risk of bias tool^1^

| **Domain** | **Questions** |
| --- | --- |
| 1. **Patient selection:** | |
| - Signalling questions (Y/N/Unclear) | Was a consecutive or random sample of participants enrolled? |
|  | Was a case-control design avoided? |
|  | Did the study avoid inappropriate selection criteria? |
|  | Were inclusion/exclusion criteria clearly reported?^1^ |
| - Risk of bias (High/low/unclear) | Could the selection of patients have introduced bias? |
| - Concerns regarding applicability (High/low/unclear) | Are there concerns that the included participants do not match the review question? |
| 1. **Hemoglobin measurement:** | |
| - Signalling questions (Y/N/Unclear) | Were the hemoglobin results interpreted without knowledge of the ferritin results? |
|  | If a threshold was used, was it pre-specified? |
| - Risk of bias (High/low/unclear) | Could the measurement or interpretation of hemoglobin have introduced bias? |
| - Concerns regarding applicability (High/low/unclear) | Is there concern that the measurement of hemoglobin, its conduct, or interpretation differ from the review question? |
| 1. **Ferritin as a reference standard:** | |
| - Signalling questions (Y/N/Unclear) | Is the measured ferritin value likely to correctly classify the target condition? |
|  | Were the ferritin results interpreted without knowledge of the results of hemoglobin measurement? |
| - Risk of bias (High/low/unclear) | Could the measurement of ferritin, its conduct, or interpretation have introduced bias? |
| - Concerns regarding applicability (High/low/unclear) | Is there concern that the target condition as defined by the ferritin results does not match the review question? |
| 1. **Flow and timing:** | |
| - Signalling questions (Y/N/Unclear) | Was there an appropriate interval between hemoglobin and ferritin measurement? |
|  | Was the blood test measured consistently across participants?^1^ |
|  | Did all participants have ferritin measured? |
|  | Were all participants included in the analysis? |
| - Risk of Bias (High/low/unclear) | Could participant flow have introduced bias? |

^1^ Question was created by the review team to address study-specific factors that were not covered in the original QUADAS-2 tool. N, no; Y, yes.

**Supplementary Figure 1** Subgroup analysis in pregnant Cambodian women (blood source and analytical method). Forest plot displaying AUC values for the discriminatory ability of hemoglobin concentration in predicting iron status (deficiency defined as unadjusted ferritin <30 µg/L). AUC, area under the curve; CI, confidence interval.


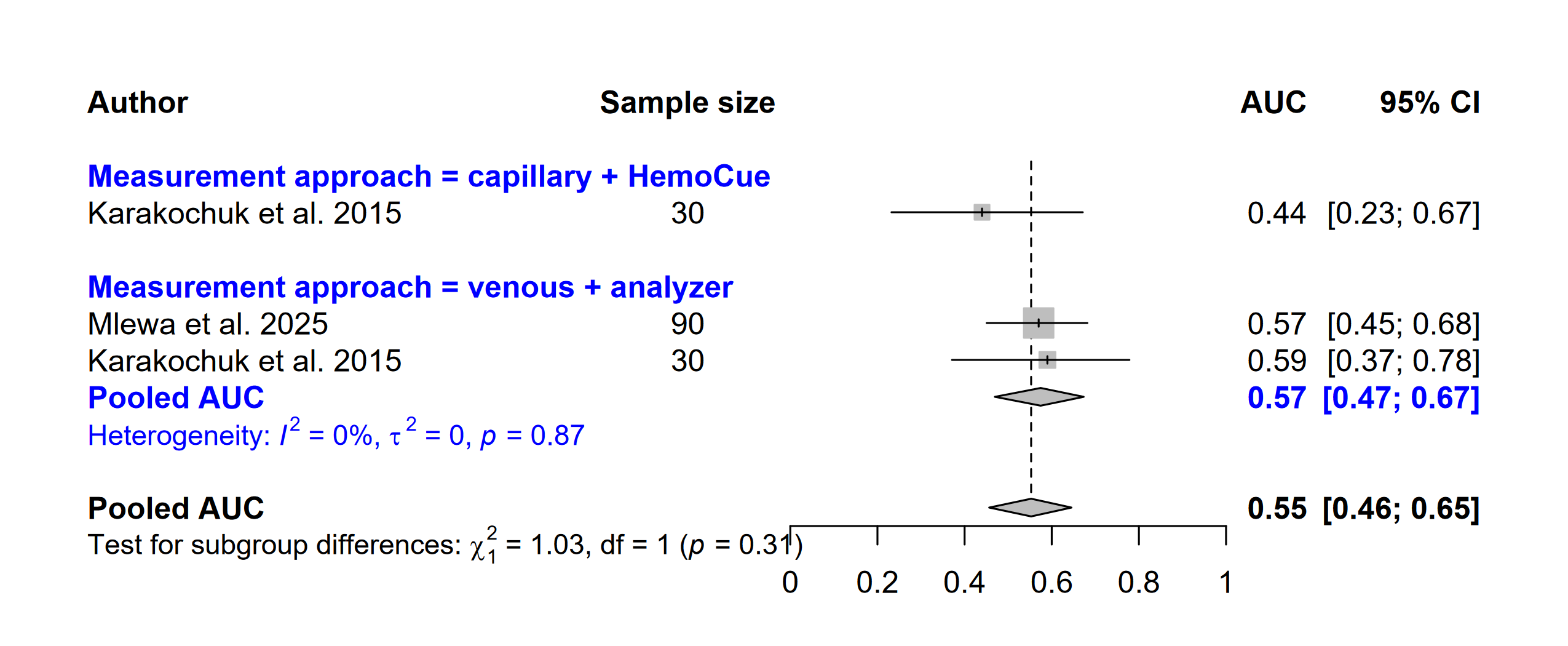


**Supplementary Figure 2** Risk of bias (ROB) assessment of included studies


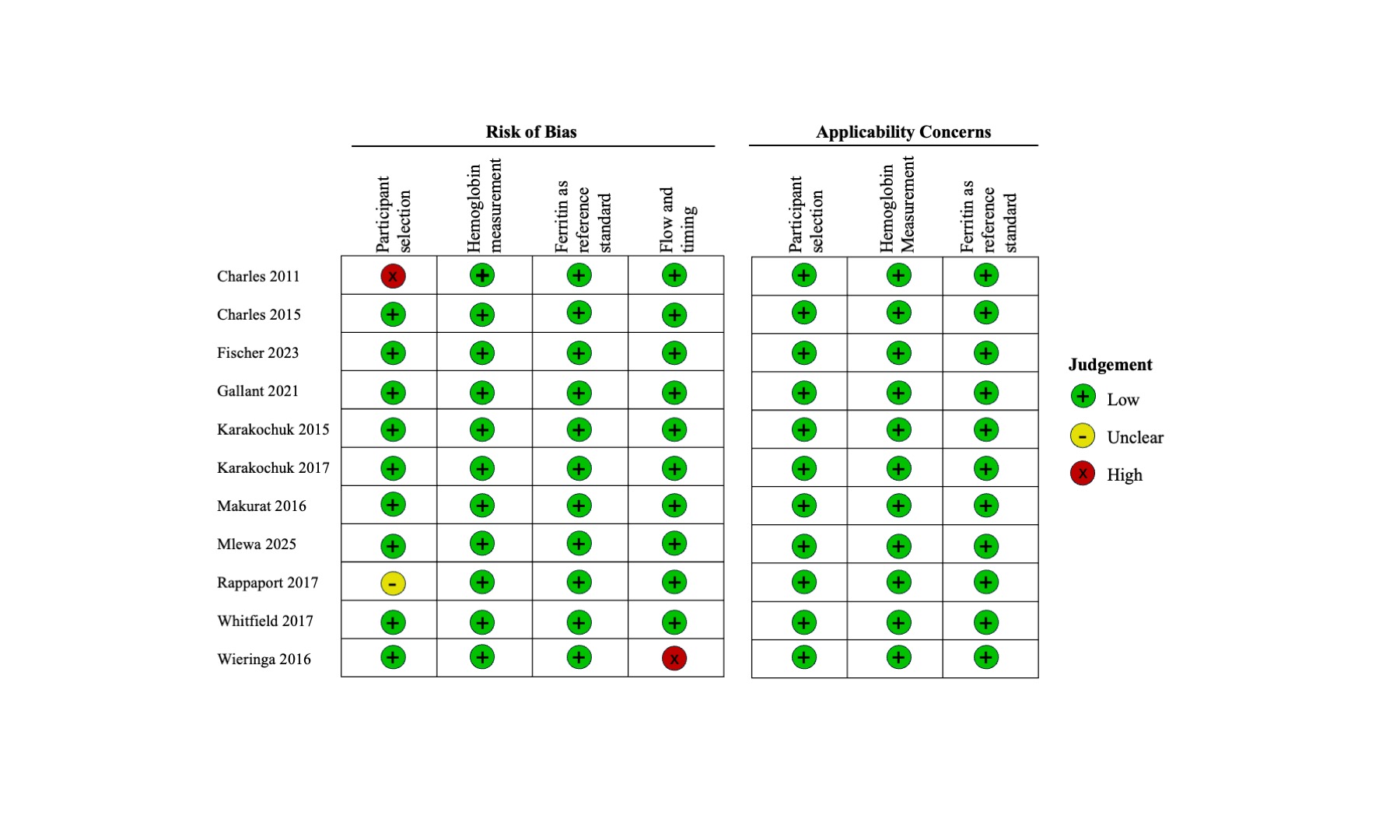

Supplement: Multimedia component 1 [file mmc1.docx]
